# Atypical Presentation of Pediatric Systemic Lupus Erythematosus Complicated by Cryptococcal Meningitis

**DOI:** 10.1155/2021/6692767

**Published:** 2021-02-11

**Authors:** Heba Ezzat Hashem, Zakaria Hamza Ibrahim

**Affiliations:** ^1^Clinical Pathology Department, Ain Shams University, Cairo, Egypt; ^2^General Surgery Department, Al-Azhar University, Cairo, Egypt

## Abstract

**Background:**

*Cryptococcus* is an opportunistic fungal pathogen that leads to life-threatening infections. Cryptococcal infections are mainly reported in HIV patients and less commonly encountered in non-HIV immunocompromised host. *Cryptococcus neoformans* (*C. neoformans*) is the most common *Cryptococcus* species causing diseases in humans which can be presented as pulmonary, meningitis, cutaneous, and/or disseminated cryptococcosis. *Case Presentation*. A 12-year-old female girl from Cairo, Egypt, presented to the pediatric hospital with signs of systemic lupus erythematosus (SLE). She had an aggressive lupus nephritis course for which corticosteroids, mycophenolate mofetil, and cyclophosphamide were prescribed, and the child gradually improved and was discharged. Two months later, the patient exhibited skin lesions involved both in her legs, massive ulcers were developed and extended rapidly through the entire legs followed by deterioration in her conscious level, and signs of meningitis were documented. Cerebrospinal fluid (CSF) examination and microbiological workup were confirmatory for *C*. *neoformans* infection, and mental and motor functions were rapidly deteriorated. Treatment with amphotericin B in addition to supportive treatment and close follow-up of the patient's medical condition result in obvious clinical improvement and patient discharge with minimal residual weakness in her legs after almost a one-month duration. After six months, the patient was brought to the emergency department complaining of repeated attacks of seizures, a lumbar puncture was performed, and culture results were again confirmatory for *C*. *neoformans*. An intensive course of antifungal therapy was prescribed which was successful, evident by resolution of the signs and symptoms of infection in addition to negative culture results and negative sepsis biomarkers. The child clinically improved, but unfortunately, gradual optic nerve degeneration and brain cell atrophy as a sequel of severe and longstanding cryptococcal infection resulted in her death after almost one year from her first attack.

**Conclusion:**

Cryptococcal infection among non-HIV patients is a rare disease but can result in advanced medical complications which may be fatal. The disease should be suspected to be reliably diagnosed. *Cryptococcus* infection can be presented as a skin lesion which, if not treated properly at an earlier time, can result in dissemination and life-threatening consequences. Amphotericin B can be used effectively in cryptococcosis management in the settings where flucytosine is not available. Signs of cryptococcal meningitis can be manifested again after a period of remission and clinical cure which signifies the latency of *Cryptococcus* in the central nervous system. The second activation of *Cryptococcus* after its latency is usually life-threatening and mostly fatal.

## 1. Background

Immunocompromised individuals including HIV, SLE, and patients on prolonged corticosteroid and immunosuppressant therapy are more prone to various bacterial, viral, and fungal opportunistic infections. One of the most important fatal fungal infections is cryptococcal infection [1–4]. *C*. *neoformans* is the most frequent cryptococcal species infect humans, and it is an encapsulated yeast microorganism, which is commonly transmitted through bird feces, plants, dust, soil, and contaminated food [2, 3]. Cryptococcosis can cause disseminated infection reaching to the lungs, central nervous system (CNS), and skin [2, 3, 5]. Dermal manifestations can present in different clinical morphologies including ulcers, acneiform papules, subcutaneous nodules, and rarely, cellulitis [4, 6].

## 2. Case Presentation

A 12-years-old Egyptian female student presented to Ain Shams University Pediatric Hospital complaining of generalized body edema, frothy urine, and proteinuria (3.7 g/day). The child was diagnosed as nephrotic syndrome, with repeated albumin infusions, and steroid therapy was initiated, but the child was nonresponding; instead, her medical condition deteriorated. Laboratory profile was in concordance with serologically quiescent SLE which included antinuclear antibody titer 1: 1280 finely speckled, elevated rheumatoid factor (RF) of 456  IU/ml (N: 0–20  IU/ml), positive SS-A/Ro  >  8.0 and SS-B/La  >  8.0 antibodies, anti-beta-2 glycoprotein IgM Ab > 100 U/ml, C-reactive protein (CRP) level 3.9 mg/l (N: 0–5 mg/l), and erythrocyte sedimentation rate (ESR) 51 mm/hour (N: 0–10 mm/hour). The serologic tests that were negative included anti-dsDNA Abs, complement C3 level 154 mg/dl (N: 106–194 mg/dl), complement C4 level 38 mg/dl (N: 19–50 mg/dl), cyclic citrullinated peptide IgG, cryoglobulin, serum immunofixation, Scl-70 scleroderma, Smith, RNP, cardiolipin antibodies, and lupus anticoagulant. Urine studies at the time revealed 3 + proteinuria, no hematuria or pyuria, a creatinine level of 0.93 mg/dl, and eGFR > 60 ml/min/1.73 m^2^. The diagnosis of SLE with nephrotic presentation was established, upon which the child received a medical regimen for lupus management including corticosteroids, mycophenolate mofetil, and cyclophosphamide which resulted in patient clinical improvement.

**Figure 1:**
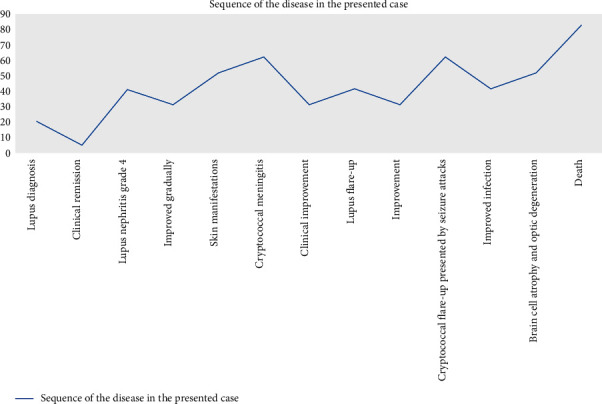
A simplified chart representing the sequence of the disease in our case.

Two months later, the child was admitted complaining of poor appetite, nausea and vomiting, tiredness, swollen ankles, and shortness of breath, and deteriorated renal functions were evident in her laboratory profile: creatinine increased to 1.92 mg/dl, 4 + protein on urinalysis, worsening urine protein to creatinine (PC) ratio of 1.9 (N: 0.0–0.1), and 24-hour urine protein of 1170 mg/24 hours (N: 50–150 mg/24 hours). Lupus nephritis grade IV was confirmed. Therefore, the child received intermittent hemodialysis; in addition, the doses of the prescribed immunosuppressant was increased: prednisone (40 mg/day), hydroxychloroquine (6 mg/kg/day), and mycophenolate mofetil (2 g/day). The patient was kept under this protocol; she gradually improved and was discharged. A simplified chart representing the sequence of the disease in the presented case is illustrated in Figure 1.

After two months, the child manifested skin ulcers in her lower extremities which were progressive in course. Drug allergies, insect bites, and family history were excluded. First, these lesions were nonwell-demarcated, erythematous, painful on palpation, nonpruritic with increasing local edema, and bullae formation. A few days later, lesions spread to involve entire her legs up to the groin area, the bullae began to coalescence together, ruptured, and a huge amount of serous discharge was drained from the lesions; no corresponding lesions appeared on the face, trunk, or upper extremities, and fever was not present at first, but with worsening of the lesions, the patient became feverish, hematuric, anemic, and hypotensive with deteriorated general condition; hence, she was admitted for the 2^nd^ time.

At the time of admission, laboratory profile revealed hemoglobin 8.2 g/dL, white blood cell (WBC) count 10,800/mm³, platelet count 129,000 cells/mm³, serum aspartate transaminase (AST) 10 U/L, serum alanine transaminase (ALT) 17 U/L, CRP 12 mg/L, and ESR 60 mm. Upon worsening the skin lesions, bacterial skin infection was suspected; so empirical treatment with amikacin and cefepime was started and the dose of prednisone decreased to 30 mg/day; microbiological cultures revealed no microorganism growth after 48 hours of incubation. Upon suspicion of bacterial cellulitis and due to the lack of improvements of the skin lesions, vancomycin was added to the treatment regimen.

**Figure 2:**
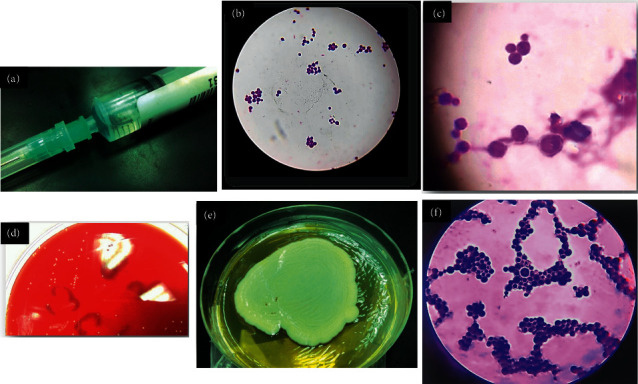
(a) Clear colorless CSF sample; other etiological causes of meningitis especially viral meningitis versus fungal meningitis must be differentiated, specifically in the presence of clear colorless CSF samples with low cell count and minimal changes of CSF parameters. (b) Spherical budding yeast in the CSF; it should be differentiated from the elliptical budding yeast cells of *Candida* spp. which is more commonly encountered during CSF examination as a fungal cause of meningitis. (c) Gram-stained film; direct film from CSF shows spherical budding yeast cells with surrounding translucent haloes due to capsular structure. (d) Blood agar shows small white colonies, after 72 hours of incubation. (e) Sabouraud dextrose agar (SDA) shows pasty colonies that were confirmed to be *Cryptococcus neoformans*. (f) Gram from culture shows spherical yeast cells with surrounding translucent haloes.

After three days, the patient medical condition deteriorated rapidly. She complained of severe headache, altered memory, continuous agitation, neck rigidity, stiffness, photophobia, and total loss of appetite; so, meningitis was suspected; hence, lumbar puncture was performed, and a CSF sample was sent for cytological examination in addition to culture procedures. The sample was clear colorless on physical inspection (Figure 2)(a), and cell count was 25 cells/HPF.

**Figure 3:**
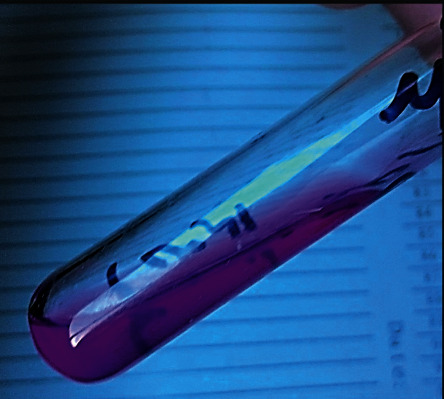
Biochemical reaction; the urease test was positive.

**Figure 4:**
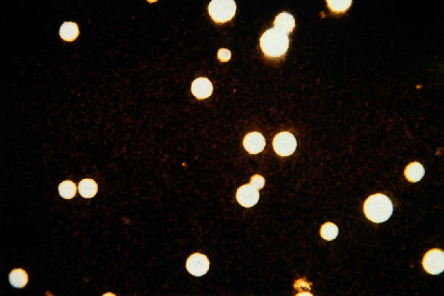
*Cryptococcus* as shiny spherical budding in a dark background.

Direct Gram examination of CSF showed spherical budding encapsulated yeast cells surrounded by translucent hallow around the cells (Figures 2(b) and 2)(c). CSF chemical examination revealed protein level 74 mg/dL (normal range 15–60 mg/dL) and glucose levels 41 mg/dL (normal range 50–80 mg/dL). The sample was cultured on aerobic blood agar, anaerobic blood agar, chocolate, and MacConkey agar; the plates showed delayed growth at the end of the third day (72 hours incubation according to CSF standard operating procedures (SOPs) of our microbiology lab). It was a minute whitish milky colony, which was highly suggestive of fungal infection, confirmed by further subculture, the sample on Sabouraud's dextrose agar (SDA) plates (Figures 2(d)–2(f)). Indian ink stain and positive biochemical reactions were confirmatory for the diagnosis of *Cryptococcus* infection (Figures 3 and 4). Additionally, antifungal susceptibility testing was performed which was positive for both amphotericin B and flucytosine.

Despite that, the child manifested obvious clinical signs and symptoms of meningitis. Magnetic resonance imaging (MRI) brain with contrast showed findings within normal limits, and blood culture was negative for microbiological growth after 7 days incubation.

Based on the available laboratory results, treatment for disseminated cryptococcosis was initiated immediately upon laboratory notification, prednisone was further decreased to 20 mg/day, and fluconazole was discontinued. Amphotericin B was started at a dose of 0.7 mg/kg/day with an infusion time of 6 hours, since the patient had repeated reactions (fever, chills, and nausea) with a shorter infusion time. Concerning flucytosine, which is indicated as the drug of choice in cryptococcal meningitis treatment, it was not used for treatment of our presented case due to financial aspects and nonavailability in our country, Egypt. Repeated lumber tapping was performed to relieve persistent increased intracranial tension as a consequence of severe meningitis; in addition, plasmapheresis was performed to remove lupus autoantibodies.

**Figure 5:**
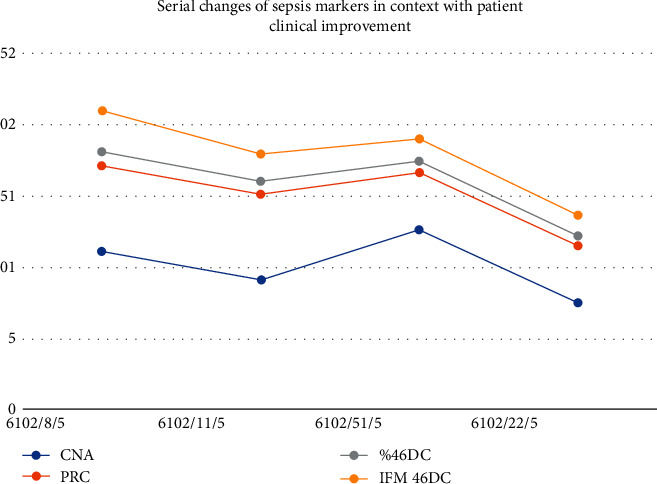
Laboratory course of the present case and serial changes of sepsis markers in context with patient clinical improvement upon administration of amphotericin B at the time of meningitis clinical manifestation; the patient improved clinically and discharged after almost one month with minimal residual weakness in her lower limbs. The nCD64 test was available in our hospital as a sepsis biomarker, and it was performed for our case for follow-up purposes.

**Figure 6:**
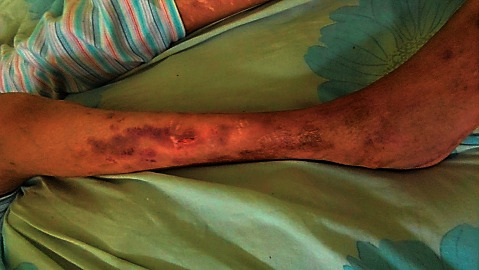
Patient developed extensive skin ulceration with serous oozing from both lower limbs; this image was taken upon her clinical improvement on amphotericin B. The local edema resolved leaving the skin redundant, cracked, and the oozing discharge was first serous-like followed by purulent discharge then pus draining stopped.

The child was subjected for further workup to rule out pneumonia and other organs affection, and for follow up purpose, chest X-ray was ordered, which revealed normal findings, ESR, CRP, complete blood count (CBC), and sepsis marker, neutrophil CD64. These laboratory tests were performed every 3 days on a routine base for monitoring the medical progress which showed dramatic gradual improvement (Table 1 and Figure 5); in conjugation with clinical signs and symptoms improvement evident by regaining her mental and motor skills, besides healing of the leg ulcers, pus draining stopped and swelling disappeared (Figure 6). In this setting, a lumbar puncture was intended to be performed upon this obvious clinical and laboratory improvement preparing the patient to be discharged, but the child refused at her own request and was discharged on her demand after almost 30 days since amphotericin B infusion was first prescribed.

At the time of discharge, the patient had no pulmonary sequelae, but she had residual weakness in her lower limbs. The laboratory results at the time of discharge were hemoglobin 9.2 g/dL, WBC 16.700 cells per mm³, platelet 303.000 cells per mm³, AST 10 U/L, ALT 4 U/L, CRP 6 mg/L, and ESR 53 mm/h. The child was instructed to continue fluconazole (400 mg/day) at home for 8 weeks and then reduced its dose to 200 mg/day for 1 year. And she was weaned gradually from corticosteroid therapy.

One month later, the child was brought to the emergency department complaining of severe headache, projectile vomiting, and blurred vision. She was admitted for the 3^rd^ time. An MRI brain was ordered, and a lumbar puncture was performed urgently to relieve intracranial tension, and for chemical, cytological, and microbiological analyses, all were negative for infection except for CRP slightly positive (12 mg/L). So, lupus flare-up was suspected, and pulse steroid therapy was started, in addition to antifungal (fluconazole) and antibiotic (amikacin and cefepime) coverage. The patient was subjected for plasmapheresis; upon it, she manifested dramatical improvement and discharge. She started to attend the immunotherapy clinic every two weeks for follow up purpose; she did well for 5 months and regained her functions except for difficulty in talking and walking abilities as she could not walk without support; so, the mother was advised to bring her for regular physiotherapy sessions every week.

**Figure 7:**
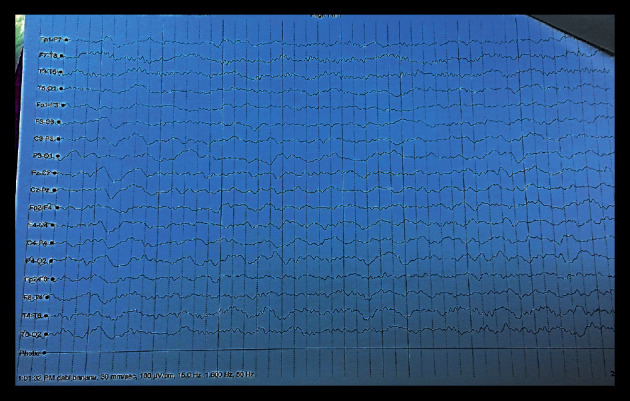
EEG at the 4^th^ admission, seizures, and other CNS manifestations in patients who had previous cryptococcal meningitis is very essential to be distinguished; it is due to reactivation of latent infection or due to seizures attributed to other medical causes. For the present case, EEG showed normal electric activates without focal localization.

Five months later, the patient complained of shivering in her legs and hands followed by seizures attacks which were gradually increasing in frequency and duration till her mother brought her to the E/R. She was admitted for the 4^th^ time. Phenytoin was prescribed, initial dose 2.5 mg/kg/dose twice a day and increased gradually to maximum 5 mg/kg/dose twice daily, and intensive work up profile was conducted searching for the cause of the seizures which included electroencephalogram (EEG), MRI brain, full lab evaluation, and CSF chemical analysis and microbiological examination. EEG revealed normal electric impulses without focal activities (Figure 7). MRI brain showed mildly dilated 3rd ventricles, CSF chemistry analysis revealed protein level 82 mg/dL (normal range 15–60 mg/dL), and glucose levels of 35 mg/dL (normal range 50–80 mg/dL). Upon receiving the sample in our microbiology lab, the sample physical criteria were very similar to the previous one, clear colorless, and cell count was 15 cell/cm^3^. A direct wet film examination was positive for budding yeast cells which were suspected and notified immediately to the pediatric team, and protocol of treatment was decided rapidly. CSF culture results were again confirmatory for *C*. *neoformans*.

**Figure 8:**
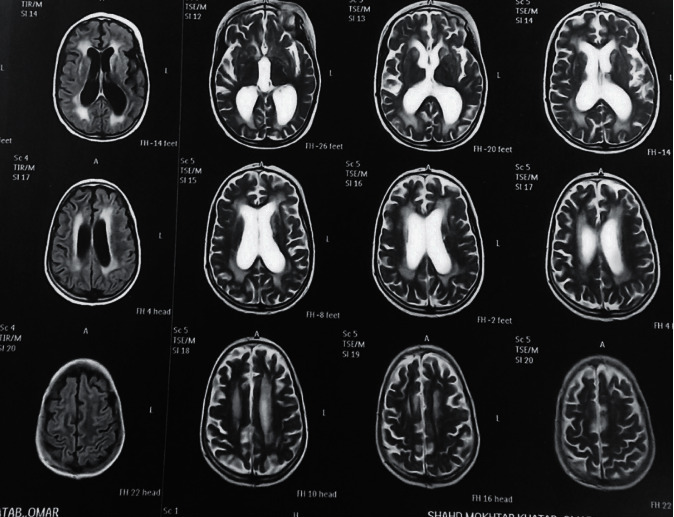
MRI brain performed at the time of 3rd admission; dilated third ventricles were evident due to brain cell atrophy; at this occasion, signs of increase intracranial tension were negative which excluded it as a cause for cerebral ventricles dilatations.

An intensive course of antifungal therapy was initiated which included amphotericin B (0.7 mg/kg/day) with an infusion time of 6 hours, and fluconazole. Resolution of patient signs and symptoms of infection was clinically evident in addition to negative culture results and improved laboratory sepsis parameters. The child general condition improved, but at this setting, she complained of poor visual acuity. The pediatric team immediately consulted the specialist neurosurgeon, ophthalmologist, and an MRI brain urgently performed which showed evidence of brain cell atrophy with subsequent increased intracranial ventricular sizes and evidence of optic nerve atrophy; the ophthalmologist confirmed bilateral optic nerve degeneration which was more evident on the left side (Figure 8).

After almost one month and, however, signs and symptoms of infection were resolved, cryptococcosis longstanding course with brain cell atrophy as a sequel of cryptococcal meningitis resulted in the child's death after almost one year from her first attack.

## 3. Discussion and Conclusion

*Cryptococcus* is an opportunistic fungal pathogen that leads to rare life-threatening infection. Meningoencephalitis and disseminated cryptococcosis are usually common complications reported in immunocompromised hosts [7, 8]. In the presented case, we reported a pediatric systemic lupus erythematosus patient complicated by rapidly disseminating cryptococcal infection which exhibited a latency in the child CNS followed by an attack of infection flare-up which was successfully treated, but slowly progressive brain cell atrophy due to longstanding disease resulted in her death.

Infections and sepsis are major causes of hospitalization, mortality, and morbidity in immunosuppressed patients [9]. Cryptococcosis is one of the fatal fungal infections reported mainly in immunocompromised hosts; however, several reported cases point to its incidence also in immunocompetent patients [10–12]. Cryptococcosis has a low incidence in non-HIV patients, approximately 1 : 100.000 [5, 13]. Patients with SLE are highly susceptible to infections due to the combined effects of their immunosuppressive therapy and the abnormalities of the immune system that the disease itself causes [14]. Concerning our presented case, prolonged corticosteroid therapy and immunosuppressants due to her underlying disease (SLE) were the major risk factors for disseminated cryptococcosis with poor outcomes.

The patient had a progressive renal course complicated by lupus nephritis class IV for which immunosuppressants was given. Lupus nephritis is clinically evident in 50–60% of patients with SLE [15]. Both mycophenolate mofetil and cyclophosphamide were prescribed to our case. Mycophenolate mofetil depletes guanosine nucleotides preferentially in *T* and B lymphocytes and inhibits their proliferation, thereby suppressing cell-mediated immune responses and antibody formation [16]. While, cyclophosphamide inhibits protein synthesis through DNA and RNA crosslinking [17].

By discussing *Cryptococcus* as the causative pathogen, *C*. *neoformans* is the most common strain causing infections in humans, and it is an encapsulated yeast that has been isolated from chickens' droppings and grows readily in soil contaminated with avian excreta particularly that of pigeons. In humans, it can colonize the upper airway system. Nevertheless, no animal-to-human nor person-to-person respiratory transmission has been documented [9]. Our presented case lived in low socioeconomic and hygienic status, and she denied contact with pigeon or birds. However, the fungus might have been indirectly transmitted via other sources such as vegetables, fruits, and dairy products [8].

*C*. *neoformans* generally cause three types of infections: cryptococcal meningitis, pulmonary, and cutaneous cryptococcosis [18]. The dissemination of this disease occurs when at least two noncontiguous sites are affected, which is unusual and mostly observed in HIV patients [4, 19]. In our case, only pulmonary involvement was not present while cutaneous manifestations were the first presenting symptoms followed by cryptococcal meningitis. Some studies support the importance of lumbar puncture in patients with cryptococcosis, even when CNS symptoms are not observed because asymptomatic meningitis is reported to be an early stage of the disease [20].

Systemic lupus erythematosus disease activity index (SLEDAI) was calculated for our presented case in order to be used as a predictor for SLE mortality and as a measure of global disease activity, and it was achieving high values (79) which reflect why the patient underwent into cryptococcal meningitis with poor clinical course. Eventhough, for patients with low SLE activity, the possibility of cryptococcal meningitis should not be underestimated [21].

Skin involvement is a rare presenting symptom of *Cryptococcus* infection, but it is usually a sign of disseminated disease; it may precede the systemic symptoms even eight months earlier [5, 18]. It is important to stress the rarity and polymorphism of the skin conditions, with the possible development of vesicles or blisters and the potential progression to ulceration [1, 4].

Cryptococcal cellulitis is a rare specific cutaneous manifestation, which was developed in our case. It was initially suspected to be a bacterial infection for which antibiotics were prescribed until its failure and microbiological results confirmed the fungal diagnosis. Although rare, the present case, cryptococcal cellulitis was restricted mainly to the lower body, particularly to the lower extremities, and this was reported in other rare reported cases [1, 2]. Despite that, cryptococcal cellulitis is uncommon and undistinguishable from acute bacterial cellulitis for appearance and presentation [6], but high suspicion especially in a risky patient should be considered.

More than 80% of patients manifested cryptococcal cellulitis and are expected to survive in immunocompetent status when they receive the appropriate antifungal therapy [6]. According to the current guidelines of the American Society of Infectious Diseases for disseminated cryptococcosis management, amphotericin B combined with flucytosine is recommended as the primary therapy, followed by fluconazole as a consolidation therapy [4, 18]. Flucytosine is expensive and was not available in our country; so, it was not considered for the treatment. Instead, it was replaced by the addition of fluconazole to amphotericin B regimen.

Cryptococcal laboratory diagnosis relies on three main methods: latex agglutination test, fungal culture, and direct microscopic examination [22]. In the present case, both the microscopic examination and microbiological cultures were the cornerstone for the diagnosis. *C*. *neoformans* was differentiated from other fungal species especially *Candida* spp. by the characterized morphology in the Gram-stained smear, microbiological culture characters, Indian ink stain positivity, and biochemical reactions.

Indian ink stain was positive in our case. In HIV-positive patients, its sensitivity increases up to 80% due to higher fungal loads in the CSF, while in HIV negative patients, the sensitivity is only 30–50% [8, 23]. CSF culture is considered the gold standard for cryptococcal diagnosis, but it has several diagnostic obstacles as long time is needed for fungus to grow, while time is very critical for management of meningitis cases with evolving lifetime neurological complications [24]. Besides, fungal cultures are not available in all medical facilities and therefore may not be performed. It is also associated with false-negative results, especially in cases with low fungal load, and this may be the attributed cause of negative blood culture result in our case.

Regarding management of cryptococcosis, the prolonged course (6–10 weeks) of IV amphotericin B monotherapy, together with the reduction of chemotherapy to improve immune status, was the main target. Caution was taken while reducing immunosuppressive therapy, since a rapid reduction is reported to be associated with immune reconstitution inflammatory syndrome (IRIS) in some patients [9]. There is evidence that supports the requirement for maintenance therapy with fluconazole administered as indefinite secondary prophylaxis, since the rate of recurrence exceeds 50% after apparently successful treatment [25].

Differential diagnosis of the present case must be addressed which includes besides to what was mentioned, infection versus flare-up attacks in lupus patients; several sepsis markers including CRP increase significantly in SLE patients with concomitant infection but increase only slightly or not at all in patients with a lupus flare without infection [14, 26] which is exactly encountered in our presented case. Additionally, decreased visual acuity due to increased intracranial tension (papilledema) was differentiated from decreased visual acuity due to optic nerve atrophy. For the presented case, papilledema was evident on fundus examination at the time of cryptococcal meningitis first diagnosed, while optic nerve atrophy was evident on fundus examination at the end.

Finally, we highlight the importance of clinicians' awareness about such infection among non-HIV immunocompromised patients including those who are diagnosed with autoimmune diseases. The latency of *Cryptococcus* should be considered and suspected during the management of such cases which necessitate early recognition and proper management.

For preparing Indian ink-stained film, take two drops from the examined CSF sample directly or a small part of the growing colony from the plate, mix it well with the ink, put a cover slide, and leave the slide for an hour to give a chance for the cells to be settled, and for better contrast view, lower the condenser and reduce the microscopic light and examine the film by using the lens with magnification power (40x). *Cryptococcus* will be evident as shiny spherical budding in a dark background.

## 4. Conclusion

Cryptococcal meningitis should be considered as one of the possible diagnoses in a patient with altered sensorium and neck stiffness, especially those who are immunocompromised. *Cryptococcus* infection can be presented as a skin lesion which, if not treated properly at an earlier time, can result in dissemination and life-threatening complications.

Early management of cryptococcal meningitis is associated with better prognosis and lower neurological complications. Despite that, flucytosine is being described as the best antifungal medication for cryptococcal infections. Amphotericin B still gives good results in such cases.

Negative bacterial growth from a skin lesion does not necessitate that it is a sterile lesion; it may be an atypical microorganism or microorganism that needs special media to grow or longer duration to be cultivated. Clinical pathologists should be aware of *Cryptococcus* laboratory diagnosis and how to differentiate it from *Candida* spp. Microbiological culture is a confirmatory step for *Cryptococcus* diagnosis; however, Gram-stained and Indian ink-stained films are rapid and informative diagnostic methods especially in limited resources facilities. The communication between laboratory staff members and treating medical team is very crucial for better clinical judgment specifically in a circumstance where an unusual diagnostic result is released or diagnosing a rare disease.

CSF examination may show low cell count; however, infection presents; this is commonly seen in immunocompromised patients as no adequate inflammation. Cryptococcal infection associated with CNS latency which should to be suspected; hence, meticulous follow-up is an essential step in cryptococcal management. The second activation of cryptococcal latency is usually life-threatening and mostly fatal.

## Figures and Tables

**Table 1:** Laboratory course of the present case, and serial changes of sepsis markers in context with patient clinical improvement upon administration of amphotericin B at the time of meningitis clinical manifestation.

	Hb	TLC	ANC	ALC	AMC	PLT	CRP	CD64%	CD64 MFI
08/05/2016	10.6	12.2	11.2	0.6	0.4	44	6	98.5	2.87
11/05/2016	9	10.9	9.2	1.1	0.51	81	6	91.5	1.91
15/05/2016	7.7	14.8	12.72	1.57	0.48	209	4	80.1	1.56
22/05/2016	7.2	10	7.6	1.1	0.5	238	4	68.9	1.45

Hb, hemoglobin (g/dl); TLC, total leukocytic count (X10^9^/L); ANC, absolute neutrophil count (X10^9^/L); ALC, absolute lymphocyte count (X10^9^/L); AMC, absolute monocyte count (X10^9^/L); PLT, platelet (X10^9^/L); CRP, C-reactive protein (mg/L); nCD64%, neutrophil CD64%; nCD64MFI, nCD64 mean fluorescence intensity.

## Data Availability

The data underlying the findings of this research are publicly available.

## Abbreviations

ALT: Alanine transaminase
ANA: Antinuclear antibody
AST: Aspartate transaminase
*C. neoformans*: *Cryptococcus neoformans*
CBC: Complete blood count
CNS: Central nervous system
CRP: C-reactive protein
CSF: Cerebrospinal fluid
DNA: Deoxyribonucleic acid
dsDNA: Double-strand DNA
E.R: Emergency room
EEG: Electroencephalogram
eGFR: Estimated glomerular filtration rate
ESR: Erythrocyte sedimentation rate
HIV: Human immunodeficiency virus
IgG: Immunoglobulin G
IRIS: Immune reconstitution inflammatory syndrome
IV: Intravenous
MPA: Mycophenolic acid
MRI: Magnetic resonance imaging
PC ratio: Protein creatinine ratio
RF: Rheumatoid factor
RNA: Ribonucleic acid
RNP: Ribonucleoprotein antibodies
SDA: Sabouraud's dextrose agar
SLE: Systemic lupus erythematosus
SOPs: Standard operating procedures
WBC: White blood cell.
